# Implementation of digital health technologies for older adults: a scoping review

**DOI:** 10.3389/fragi.2024.1349520

**Published:** 2024-05-07

**Authors:** Jeffrey W. Jutai, Farah Hatoum, Devvrat Bhardwaj, Marjan Hosseini

**Affiliations:** ^1^ Interdisciplinary School of Health Sciences and AGE-WELL Network of Centres of Excellence, University of Ottawa, Ottawa, ON, Canada; ^2^ School of Epidemiology and Public Health, University of Ottawa, Ottawa, ON, Canada; ^3^ School of Electrical Engineering and Computer Science, University of Ottawa, Ottawa, ON, Canada; ^4^ School of Rehabilitation Sciences and AGE-WELL Network of Centres of Excellence, University of Ottawa, Ottawa, ON, Canada

**Keywords:** ambient assisted living, aging, digital health, gerontechnology, technology implementation, scoping review

## Abstract

The critical importance of technological innovation in home care for older adults is indisputable. Less well understood is the question of how to measure its performance and impact on the delivery of healthcare to older adults who are living with chronic illness and disability. Knowing how well digital technologies, such as smartphones, tablets, wearable devices, and Ambient Assisted Living Technologies (AAL) systems “work” should certainly include assessing their impact on older adults’ health and ability to function in daily living but that will not guarantee that it will necessarily be adopted by the user or implemented by a healthcare facility or the healthcare system. Technology implementation is a process of planned and guided activities to launch, introduce and support technologies in a certain context to innovate or improve healthcare, which delivers the evidence for adoption and upscaling a technology in healthcare practices. Factors in addition to user acceptance and clinical effectiveness require investigation. Failure to appreciate these factors can result in increased likelihood of technology rejection or protracted procurement decision at the “adoption decision” stage or delayed or incomplete implementation or discontinuance (following initial adoption) during implementation. The aim of our research to analyze research studies on the effectiveness of digital health technologies for older adults to answer the question, “How well do these studies address factors that affect the implementation of technology?” We found common problems with the conceptualization, design, and methodology in studies of digital technology that have contributed to the slow pace of implementation in home care and long-term care. We recommend a framework for improving the quality of research in this critical area.

**Systematic Review Registration:** https://archive.org/details/osf-registrations-f56rb-v1, identifier osf-registrations-f56rb-v1.

## Introduction

The critical importance of technological innovation in home care for older adults is indisputable (Rogers and Mitzner, 2017; Linskell et al., 2019; Rajer and Bogataj, 2022). Less well understood is the question of how to measure its performance and impact on the delivery of healthcare to older adults who are living with chronic illness and disability (Matthew-et al., 2016). Knowing how well digital technologies, such as smartphones, tablets, wearable devices, and Ambient Assisted Living Technologies (AAL) systems “work” should certainly include assessing their impact on older adults’ health and ability to function in daily living. However, it should not be assumed that, because a technology produces clinical benefits and is acceptable to the user, it will necessarily work in the sense that it provides solutions for the needs of users and healthcare facilities that will be adopted and implemented.

Technology implementation has been defined as a process of several planned and guided activities to launch, introduce and maintain technologies in a certain context to innovate or improve healthcare, which delivers the evidence for adoption and upscaling a technology in healthcare practices (van Gemert-Pijnen, 2022). For digital technologies to be successfully implemented in delivery of healthcare and social services to older people, factors in addition to their user acceptance and clinical effectiveness require investigation. Failure to appreciate these factors can result in increased likelihood of technology rejection or protracted procurement decision at the ‘adoption decision’ stage or delayed or incomplete implementation or discontinuance (following initial adoption) during implementation (Kyratsis et al., 2012).

**FIGURE 1:**
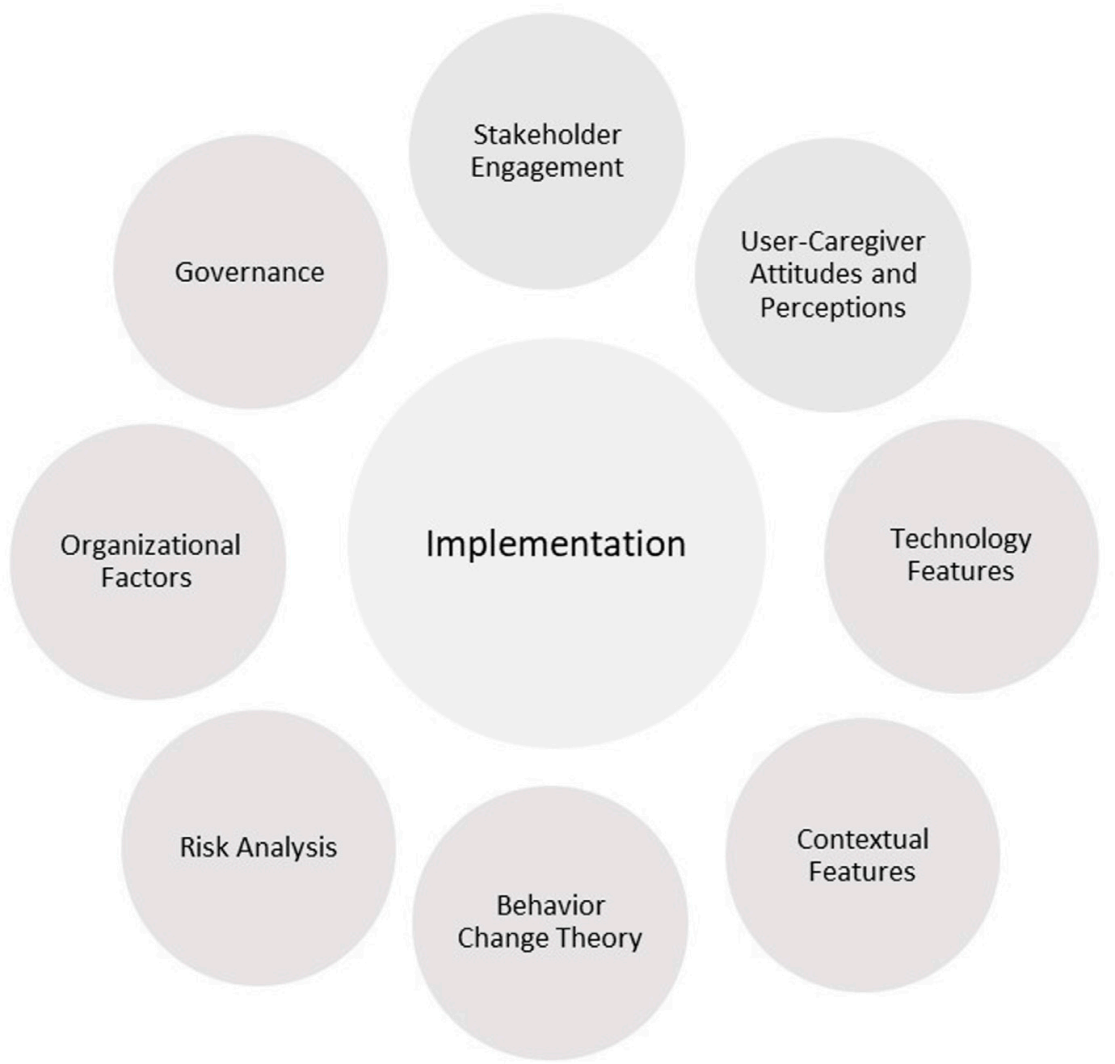
Categories of factors affecting implementation of digital technologies.

Categories of factors that are proposed to affect the implementation of healthcare technologies have been identified in the literature (Kyratsis et al., 2012; Keyworth et al., 2018; van Gemert-Pijnen, 2022) and are depicted in Figure 1. Some categories, such as Governance, Organizational Factors, and Risk analysis, may be more applicable to some residential settings (e.g., long-term care facilities or retirement communities) than others (e.g., community-dwelling older adults). For example, issues of governance and care facility management would have lesser importance for older adults who consume technologies in their private homes and apartments but significant importance for agencies that purchase and maintain technologies for older adults who are under their care.

*Stakeholder engagement* is regarded as crucial for ensuring that a technology realizes the values of end users and other stakeholders and identifying issues for implementation (van Gemert-Pijnen, 2022). *Behavior change theory* is recognized as an important ingredient (Keyworth et al., 2018). Types of technology interventions that have been shown to be effective include reminders and alerts and computer-generated feedback. The most targeted behavior is adherence to clinical prescriptions and prescribing behaviors. Behavior change techniques include instruction on how to perform the behavior, feedback on behavior, prompts and cues, demonstration of the behavior, reducing negative emotions, social comparison, and problem solving. *Contextual features* include practice and workload considerations. *Features of the technology* include pilot testing before wide-scale usage, an iterative modification approach, and the ease of use of the technology. The *attitudes and perceptions* of older adult users and their caregivers, who include healthcare providers, are important for adoption and use. *Organizational factors* are also critical for successful implementation (Kyratsis et al., 2012). Technology researchers and developers should recognize that the key motivator for adoption decisions by healthcare organizations is finding solutions to problems. Among types of knowledge, scientifically produced research evidence has the highest priority for judging technology effectiveness. It is often combined with experiential (“how-to”) knowledge to evaluate the appropriateness of technology for a particular setting. *Governance*, by way of establishing vision, leadership, policy, and accountability, is essential for sustaining implementation through appropriate planning, commitment of staff, manageable workload, and positive attitudes (van Gemert-Pijnen, 2022).

There will necessarily be variation in implementation performance since different agencies, whether they be households, healthcare facilities, or healthcare systems, will not respond in these categories in the same way (Goggin, 1986). Technology researchers and developers should be aware of this variation and consider how they might assist potential adopters to do implementation planning within their areas of competence and expertise.

A final set of factors pertains to *risk analysis*, which helps us understand and prepare responses for the potential risks associated with adopting and implementing the new technologies. Brown and Osborne (Brown and Osborne, 2013) identified the key elements for analysis and classified risks as follows. The locus for c*onsequential* risk is the individual and refers to direct risk to the user of the digital health service. For example, deviation from established approaches to home care for older adults may introduce emotional distress and risks to physical health. The locus for *organizational* risk is the service agency and its staff. The risk here involves vulnerable individuals remaining living independently for longer than they might have been able to and the implications for the organizational or professional reputation and/or legitimacy and sustainability of the service agency. For example, managers and staff tend to be risk averse and may tend toward concealing errors instead of identifying and learning from them. The locus for *behavioral* risk is the community of interest and involves risk to the stakeholders surrounding a service and/or the wider community. For example, digital health technologies, while offering a more appropriate response to the needs of community-dwelling older adults, can lead to risks to other people in the community, such as distress to uncomprehending relatives and neighbors. Implementation is undermined by failure to acknowledge and discuss these risks.

As an important first step toward understanding the state of knowledge on this topic, we performed a scoping review. The aim was to characterize the research available to address the question, “How well have research studies on the effectiveness of digital health technologies for older adults addressed factors that affect the implementation of these technologies?” In our analysis, we looked for evidence that researchers considered the implementation factor categories described above in their studies.

## Methods

### Study design

We conducted a scoping review of the peer-reviewed literature with an unlimited publishing time limit. As described by Arksey and O’Malley (Arksey and O’Malley, 2005) and Levac et al. (Levac et al., 2010), the use of scoping reviews was determined to be the most appropriate approach to collate a wide range of evidence and identify research gaps in the literature. The findings can be used for mapping a complex area of investigation and informing future research. The population of interest is older adults living with chronic health conditions and disabilities. In this review, we did not restrict the range of digital health technologies and settings for their application, as we were interested to learn if studies addressed factors for digital health technology implementation, whether an older adult was living at home in the community or in long-term care or similar facility. The potential range of devices includes ambient assisted living (AAL) systems, wearable sensors, smart everyday objects, environmental sensors, and social assistive robots that are intended to be used by older adults, their caregivers, and healthcare providers (Cicirelli et al., 2021).

The research question was composed based on a lack of consensus in the research literature on the most appropriate set of indicators for the successful implementation of digital health systems in all settings that matter. It should be noted that we did not evaluate methodological quality of included studies, in accordance with the convention for scoping reviews. A detailed search strategy for peer-reviewed literature was developed prior to conducting any searches. The review protocol was designed and conducted in accordance with the Preferred Reporting Items for Systematic Reviews and Meta-Analyses extension for Scoping Reviews (PRISMA-ScR) (RRID:SCR_018721) guidelines and was registered with Open Science Framework (RRID:SCR_003238) to increase research transparency and prevent any duplication efforts as per best practice guidelines.

### Eligibility criteria

We considered all peer-reviewed journal articles related to technologies for assisted living for older adults (aged 60+ years) and published in English. This age group was selected as the definition of ‘older adults’ because in most contemporary Western countries, 60 or 65 is the age of eligibility for retirement and old-age social programs (OECD, 2021). We did not consider any grey literature reports. A complete list of the eligibility criteria is shown in Table 1. To be included in this review, the authors of the original papers did not have to explicitly name “implementation” as an outcome or objective of the study. The reviewers included all studies that reported outcome measures relevant to the effectiveness, feasibility, and implementation of digital technologies as noted in the introduction to this article.

**TABLE 1:** Scoping review search methodology: eligibility criteria.

Eligibility criteria	Exclusion criteria
Unlimited timeframe. Specific timeframe will be concluded once all articles have been found	Newspaper articles, working papers, conference papers, editorials, or book chapters
Worldwide data	N/A
Original research; unpublished trials, any other Reviews	Did not describe original research
Evidence on indicators of successful technology implementation among elderly people	N/A
Available in English	In languages other than English

## Search strategy

The technical information about the search strategy is presented in Appendix. A systematic search of the following five academic databases was conducted to identify relevant peer-reviewed results: Ovid MEDLINE/PubMed, Scopus, Ovid Embase, Ovid PsycINFO and Ovid Cochrane Library (Cochrane Central Register of Trials). The search strings used for the academic databases (available on request from the authors) were developed with guidance from a university librarian with expertise in the health sciences. This search of electronic databases was conducted using only English search terms. All results retrieved by the search were imported into Covidence (RRID:SCR_016484; Veritas Health Innovation, Melbourne, AU, 2020) a web-based software for systematic reviews, and duplicates were automatically removed.

## Selection of articles for review

**FIGURE 2:**
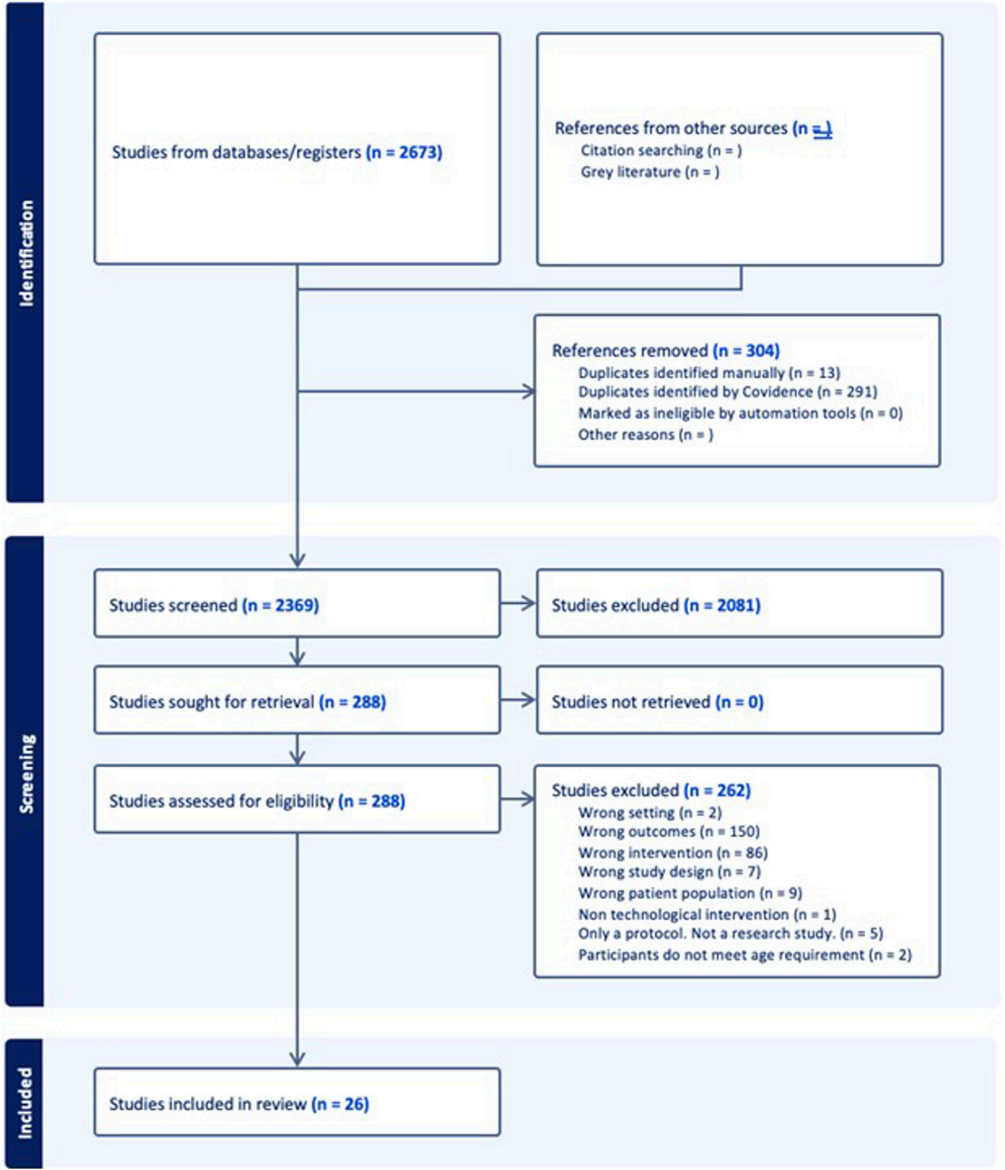
PRISMA flow diagram of systematic search of peer-reviewed and grey literature.

Two reviewers (FH and DB) screened all peer-reviewed results using Covidence. The screening of search results from the electronic academic databases occurred in two phases. First, two reviewers (FH and DW) independently screened the title and abstract of each article using the predefined eligibility criteria; any disagreements were resolved via consensus. Next, both reviewers screened the full texts of potential articles for eligibility by both reviewers. Disagreements were also resolved by discussion and/or consultation with a third reviewer (JJ) when necessary. All articles that remained after full text screening were included in the study. Figure 2 summarizes study selection process for peer-reviewed and grey literature, based on the PRISMA-ScR reporting guidelines.

A total of 150 studies were dropped at the full-text screening phase as “wrong outcomes” and 86 as “wrong intervention”. Our selection criteria did not exclude outcome measures and digital health technology interventions based upon lists of examples because we did not want to risk overlooking any promising studies. Studies that were screened out for the reasons listed above included those that described outcome measures and interventions that were irrelevant for implementation, such as bio-signal characteristics and electronic medical records, respectively.

### Data extraction and charting

Following the screening of results, three reviewers (FH, DB, MH) extracted from each article. The extracted data included author and publication year, publication type, data collection period, population and key results related to the research question. Four reviewers (FH, DB, MH, JJ) completed validation of the extracted data. We subsequently grouped the results by outcome measure and relevancy.

### Data analysis

As scoping reviews typically do not include an assessment of methodological limitations or the potential for evidence bias (Munn et al., 2018), we elected to focus our analysis on the implications for future research design, rather than the practical applications of our findings.

## Results

Table 2 presents essential information from the 26 selected articles, which includes the study population, nature of the technology intervention, the outcomes that were assessed, and implications of the study findings for technology implementation. Five of the articles were reviews (scoping or systematic). The populations researched in these articles were overwhelmingly community-dwelling older adults (*n* = 17), but also long-term care and nursing homes (*n* = 4), retirement community or village (*n* = 3), setting not specified (*n* = 1), and implementation stakeholders (*n* = 1). The categories of interventions were information and communication technologies (*n* = 7), wearable sensors (*n* = 2), ambient assisted living technologies (*n* = 6), assistive robots (*n* = 5), interventions to overcome barriers to using technologies for aging in place (*n* = 1). Some articles discussed several different categories (*n* = 5). The domains in which outcomes were assessed spanned a wide range at the levels of users (or residents), healthcare providers, and healthcare facilities. Most of the studies used measures of user acceptance and intention to use technology (derived from the Technology Acceptance Model and its variants) (*n* = 9). Note that only nine of these studies explicitly discussed the relevance of the findings for implementation.

**TABLE 2:** Details from the selected articles.

Article	Population	Intervention	Outcomes assessed	Implementation implications
^a^Bail et al., 2022 (Bail et al., 2022) (systematic review)	older adults in long-term care and nursing homes	health information technologies for electronic health record, medication management, skin management, and communication (e.g., telehealth)	facility-level outcomes, nurse outcomes, and resident outcomes for acceptability, satisfaction user perceptions, worker time, timeliness of care, and quality of care	the probability of implementation success is higher when technology systems are co-designed with residents and staff; most of the studies reported technology problems and maintenance issues; the functionality of the technology was poorly communicated and did not meet user needs and expectations; rarely was the same outcome assessed across studies; studies seldom captured the complexity and relatedness of resident care needs
Berquist et al., 2020 (Bergquist et al., 2020)	community-dwelling older adults	smartphone app–based self-tests of physical function	usability measures	high error rates due to users misunderstanding instructions
*Bieryla et al., 2013 (Bieryla and Dold, 2013)	older adults living independently in a senior living community	Nintendo Wii Fit for improving balance	Berg Balance Scale, Fullerton Advance Balance Scale, Functional Reach test, and Timed Up and Go test	the study did not actually include training in the participants’ homes
*Braspenning et al., 2022 (Braspenning et al., 2022)	three stakeholder groups involved in the implementation process of lifestyle monitoring (informal caregivers, healthcare professionals, and healthcare managers)	technology for ambient assisted living	interview guide based on normalization process theory (NPT) constructs (coherence, cognitive participation, collective action, and reflexive monitoring)	barriers to implementation were a perceived inflexibility in how the technology should be used and integrated with organizational workflows; lack of a clear business case for engaging with healthcare managers; and perceived unreliability of the technology
Broadbent et al., 2015 (Broadbent et al., 2015)	older adults residing in a retirement village hospital and rest home setting and care staff	multiple healthcare robots	quality of life, depression, and dependency (mobility, activities of daily living, and behaviour)	no safety concerns; staff were more positive toward robots than residents
*Cavallo et al., 2015 (Cavallo et al., 2014)	community-dwelling older adults with dementia	technology for ambient assisted living	unvalidated 5-point rating scales about the technology’s usefulness, obtrusiveness, and acceptability, from a multidisciplinary team of clinicians, engineers, psychologists, and therapists	authors stressed the importance of extensive consultation with stakeholders on technical, ethical, legal, clinical, economic, and organizational implications of technology implementation
Choukou et al., 2021 (Choukou et al., 2021) (scoping review)	community-dwelling older adults	technology for ambient assisted living	perceived usefulness, ease of use, intention to use, and user acceptance	authors concluded that the methodological quality of research in this area was poor (only one study evaluated all four aspects of the Technology Acceptance Model); need for studies that use a comprehensive evaluation framework that considered the needs and preferences of intended users at each stage of technology development
Fan et al., 2017 (Fan et al., 2017)	older adults in long-term care and nursing homes	socially assistive robots (SARs)	user’s acceptance and intention to use new technology based on performance expectancy, effort expectancy, attitude toward using technology, and self-efficacy; level of enjoyment and interest	Authors recommended studies of long-term effect of SARs, including misuse of robots, decreased human contact, loss of control, loss of privacy, and feelings of objectification
Fiorini et al., 2021 (Fiorini et al., 2021)	older adults living in long-term care and nursing homes	robots that provide functional assistance (ASTRO robot)	attitudes and beliefs in the ability of the robot to address primary needs; concerns about stigma and replacement of human care	Implementation challenges included the capability of robots to navigate dynamic variation in the physical and social/cultural environments in the longer term, to detect and manipulate a wide variety of objects in different contexts, to act autonomously, and to interpret human emotions and react appropriately in social situations
Gettel et al., 2021 (Gettel et al., 2021) (scoping review)	community-dwelling older adults	software apps, augmented and virtual reality, care robots, home monitoring systems, intelligent cognitive assistants, and wearable activity monitors and cameras	measures of behavior, working memory and physical activity	barriers to adoption and implementation included lack of experience with technology, difficulties learning to use technology, privacy concerns, and fears that technology (e.g., social robots) would lead to social isolation; potential users need education and training; implementation studies are badly needed
Law et al., 2019 (Law et al., 2019)	community-dwelling older adults with mild cognitive impairment (MCI) or early dementia	home-based healthcare robot	perceived usefulness	implementation challenges included technical problems with the robot and lack of research studies with longitudinal designs and comparison of different robots
Lesauskaitė et al., 2019 (Lesauskaitė et al., 2019)	community-dwelling older adults and geriatric in-patients	computers, the internet, smartphones, and fall detectors	self-report questionnaire on the knowledge, readiness to use, and use of technologies	smartphones were less stigmatizing than non-digital technologies; privacy concerns about smart home technology were inversely correlated with user health needs
McMahon et al., 2016 (McMahon et al., 2016)	community-dwelling older adults	wearable physical activity monitors (Fitbit One)	questionnaire based on the Technology Acceptance Model (TAM)	the technology was found to be easy to use, useful and acceptable; authors recommended that studies compare several types of monitors, and measure emotional satisfaction and health benefits
Moyle et al., 2018 (Moyle et al., 2018)	dementia patients in long-term care	PARO robotic seal	motor activity and sleep patterns (SenseWear^®^ armband)	participants did not tolerate wearing the armbands; devices were often unreliable in their recording; care staff should monitor adherence and remind residents about wearing devices appropriately
*Moyle et al., 2021 (Moyle et al., 2021) (scoping review)	community-dwelling older adults with dementia	technology for ambient assisted living	various measures of technology effectiveness	authors recommended that evaluation of the technology should occur only when it is at a level of sufficient development, to avoid ongoing and disruptive technical issues
Neal et al., 2021 (Neal et al., 2021) (systematic review)	community-dwelling adults with a diagnosis of dementia or with MCI	digital technology (if it was inherently dependent on any electronic device that comprised, or interfaced with, any kind of computer)	self-management and social participation	study authors made surprisingly few statements about implementation; identified implementation factors were enjoyment from technology use and complexity or limited functionality of technology; the availability of high-quality evidence in this field does not seem to have significantly progressed from previous reviews
Orellano-Colón et al., 2016 (Orellano et al., 2016)	community-dwelling older adults	a wide variety of technology devices for aging in place, including digital health technologies	user-perceived challenges, barriers or obstacles for using technology devices	lack of awareness and information about technology, cost, limited coverage of technology by healthcare plans, and perceived complexity of technology
Orellano-Colón et al., 2020 (Orellano et al., 2020)	community-dwelling older adults	intervention to overcome barriers to using technologies for aging in place	acceptability, effectiveness, physical and mental health, psychosocial impact, and self-efficacy	self-management and behavioural change techniques for technology users can facilitate implementation
*Peek et al., 2017 (Peek et al., 2017)	community-dwelling older adults	a wide variety of technology devices for aging in place, including digital health technologies	reasons for device ownership and frequency of use, and attitudes and perceptions about these devices	factors affecting implementation included: favorable or unfavorable beliefs concerning the reliability, lifespan, power consumption, and costs of purchase and maintenance of technology; positive and negative consequences of using technology for users and caregivers; self-efficacy for using technology; user’s social network, social agencies, and compatibility of the technology with the user’s physical environment
*Reeder et al., 2013 (Reeder et al., 2013)	older adults living in a retirement community	technology for ambient assisted living	self-report standardized measures of physical mobility, psychosocial health, and cognitive health, and fall tracking	authors reported significant implementation challenges with the sensor-based monitoring system; recommended that study designs use mature and reliable technology, provide adequate resources for installations, and ensure that participants are informed in advance that there may be technical problems
Sánchez et al., 2019 (Sánchez et al., 2019)	community-dwelling older adults	technology for ambient assisted living	attitudes and perceptions	implementation challenges included costs, loss of autonomy and personal dignity, and a preference for human care; participants were not concerned about privacy
*Sautter et al., 2021 (Sautter et al., 2021)	older adult with mild and advanced dementia	touch-screen computer applications to enhance social connection, facilitate entertainment, and implement cognitive training	frequency counts of challenging behaviors and cognition (attention, concentration, executive functions, memory, language, conceptual thinking, calculations, and orientation)	authors recommended that studies with participants who have dementia should use ongoing processes for assent and building rapport between study personnel and participants (both older adults and caregivers)
Schoon et al., 2020 (Schoon et al., 2018)	community-dwelling older adults	self-management fall prevention program using a wearable gait-speed feedback device	compliance (number of weekly gait speed measurements and reasons for not having a measurement), falls (via telephone), mobility (Timed Up and Go), and disability (Katz-15 scale)	the intervention had good technical feasibility and compliance, but it did not produce overall positive outcomes; authors recommended that future research examine all constructs of the TAM
*Selye et al., 2020 (Seelye et al., 2020)	community-dwelling older adults	sensors and software to monitor pill-taking, steps taken, time spent sleeping, and computer use in a real-world assessment of digital health technology in the homes	standardized neuropsychological test, health assessments, and daily function questionnaires	feasibility issues included technical problems with installation and in-home technology maintenance
Wang et al., 2020 (Wang et al., 2020)	community-dwelling older adults	an integrated, personalized telehealth monitoring system (steps and sleep data using a Fitbit, and gait and balance status using wearable sensors)	users’ acceptance of the system with respect to attitude, self-efficacy, perceived usefulness, perceived ease of use and behavioural intention	users found the system easy and comfortable to use and useful for improving their health, and intended to use the system in their future health management
Wu et al., 2015 (Wu et al., 2015)	community-dwelling older adults	information and communication technologies (ICTs)	attitudes toward ICTs	authors recommended that older adults get appropriate training and support in ICT use through peer-supported training, to improve their technology skills and their attitudes toward technology

^a^ Denotes that the authors made explicit reference to the relevance of their research for technology implementation.

From the list of implementation issues identified in the selected studies, we detected the following themes:

1. *Communication* (technology utilization and functionality poorly communicated to users).
2. *Context sensitivity* (e.g., need to train users in their homes; need to investigate workflow compatibility).
3. *Design* (importance of co-design with users and caregivers).
4. *Economic analysis* (e.g., cost-benefit analysis).
5. *Ethical considerations* (e.g., need for ongoing processes for assent for users with dementia).
6. *Outcome assessment* (inconsistent across studies; not comprehensive with respect to user and caregiver needs, emotional satisfaction, and health benefits).
7. *Quality of research* (weak evidence basis; poor methodology; lack of theoretical grounding; lack of cross-product comparisons; lack of longitudinal studies to analyze dynamic variations in physical, social, and cultural environments).
8. *Technology maturity and readiness* (unreliability; malfunction and maintenance concerns).
9. Unmet user needs and expectations.
10. *User education and training* (e.g., peer-supported training).

Table 3 presents an analysis of whether the selected articles referred to implementation factors presented in Figure 1. Those marked with an asterisk (*) made explicit reference to the relevance of their research for technology implementation. Most of the articles reviewed made only indirect references to implementation. The overwhelming focus of studies has been on implementation issues related to features of the technology under investigation and the attitudes and perceptions of technology users and caregivers. As previously noted the methodological rigour of these studies was weak. Very few studies used a theory-driven approach and validated methods for assessing attitudes and perceptions. We were unable to find any studies of the impact of governance and risk analysis on the implementation of digital health technologies for older adults.

**TABLE 3:** Analysis of implementation factors for the selected studies.

Category of implementation factor									
Article	Behavior change theory	Contextual features	Stakeholder engagement	Technology features	User-caregiver attitudes and perceptions	Governance	Organizational factors	Risk analysis	No. of categories assessed
^a^Bail et al., 2022		**√**	**√**	**√**	**√**		**√**		5
Berquist et al., 2020				**√**					1
^a^Bieryla et al., 2013				**√**					1
^a^Braspenning et al., 2022		**√**	**√**	**√**	**√**		**√**		5
Broadbent et al., 2016		**√**		**√**	**√**				3
^a^Cavallo et al., 2015			√	√			√		3
Choukou et al., 2021					√				1
Fan et al., 2017				√					1
Fiorini et al., 2021				√	√				2
Gettel et al., 2021	√			√	√				3
Law et al., 2019				√	√				2
Lesauskaitė et al., 2019					√				1
McMahon et al., 2016					√				1
Moyle et al., 2018		√		√	√				3
^a^Moyle et al., 2021				√					1
Neal et al., 2021	√			√					2
Orellano-Colón et al., 2016				√	√				2
Orellano-Colón et al., 2020					√				1
^a^Peek et al., 2017		√			√				2
^a^Reeder et al., 2013				√	√				2
Sánchez et al., 2019					√				1
^a^Sautter et al., 2021	√		√						2
Schoon et al., 2020				√	√				2
^a^Selye et al., 2020				√					1
Wang et al., 2020					√				1
Wu et al., 2015		√			√				2
**Category Total**	3	6	4	17	18	0	3	0	

^a^ Denotes that the authors made explicit reference to the relevance of their research for technology implementation.

## Discussion

We found that understandings about factors affecting implementation across research studies of digital health technologies for older adults varied significantly and was reflected by a wide variety of methods for technology evaluation and uneven quality of research. Most of the research studies were either focused on the smart home technology development phase or were laboratory-based evaluation studies that narrowly defined implementation in terms of technical feasibility, clinical effectiveness, and user acceptance. None of these articles examined the full range of implementation factors depicted in Figure 1 for either community-dwelling older adults or those residing in long-term care facilities.

Areas where high-quality research is particularly needed include managing risks of discontinuous, potentially disruptive innovations by health and social services (Brown and Osborne, 2013), developing culturally and linguistically appropriate technology-delivered interventions for ethnic minority older adults (Chung et al., 2016), evaluating the impacts of technology implementation on service delivery (Cucciniello and Nasi, 2013), and adoption and long-term use of technologies for health self-management (Courtney, 2008; Lee and Coughlin, 2015). In each setting in which digital health technologies may be deployed, we need to understand how they should be developed alongside the networked social relations that make them ‘work’ and pragmatically customized to meet older adults’ unique and changing medical, personal, social, and cultural needs” (Greenhalgh et al., 2015; Sánchez et al., 2015; Jutai et al., 2022).

It is unreasonable to expect that a single research study should investigate in all eight of the categories of implementation factors that we identified. We argue, though, that the quality of research in this area would be markedly improved if researchers would subscribe to a more comprehensive program logic for their studies. For example, investigations of the technical feasibility, clinical effectiveness, or user acceptance of a new technology should show how they have anticipated the most pressing concerns that might arise from the domains of behavior change, governance, organizational factors, risk analysis, and stakeholder engagement. Adherence to an accepted conceptual framework should improve the likelihood of successful technology implementation. Several published frameworks, such as NASSS (Greenhalgh and Abimbola, 2019), CeHREs (van et al., 2011), and SCIROCCO (Grooten et al., 2020) offer excellent, detailed guidance, and we encourage researchers to consider them. We recommend their use because they can inform the design of a new technology, identify technological solutions that have a limited chance of achieving large-scale, sustained adoption, help to plan the implementation of technology, and help explain and learn from implementation failures (Greenhalgh et al., 2017). These frameworks are underutilized at present, due in part to their recent development, but probably also due to a need for them to be adequately contextualized for individual technologies (Abell et al., 2023).

## Limitations

Our study did not include stakeholder consultation on the scoping review but we plan to conduct stakeholder evaluations of the provisional framework, to identify opportunities for its application across the broadest possible range of digital health technologies for older adults.

## Future directions

Our findings reinforce the view that implementation is often planned and executed only after the design of a technology has been completed. Future studies of technology development should include consideration of the eight categories of factors that can affect implementation. They should acknowledge the implications of the technology not only for individual users, but the healthcare system and society at large (van Gemert-Pijnen, 2022). We recognize that it is challenging for a single study to address all these factors, but if indeed the goal for the research is to achieve successful implementation of the technology, then researchers should be expected at least to demonstrate their awareness of the issues.

## Data Availability

The original contributions presented in the study are included in the article/Supplementary Material, further inquiries can be directed to the corresponding author.
