# Supplementary material for: Implementation of digital health technologies for older adults: a scoping review
Source: Front Aging. 2024 May 7;5:1349520. doi: 10.3389/fragi.2024.1349520 (PMC11112488; doi:10.3389/fragi.2024.1349520)
Supplement: Supplementary file 1 [file DataSheet1.docx]

**Appendix: Publication Database Search Strategy**

**Medline (Ovid) Search Strategy – 472 Results [in Covidence]**

| **#** | **Query** | **Results from 26 Jul 2022** |
| --- | --- | --- |
| 1 | exp Aged/ | 3,412,539 |
| 2 | exp Geriatrics/ | 31,205 |
| 3 | (elders or elderly or geriatric* or "gerontolog* old age" or (seniors not "high school") or (older adj3 (adult*or person* or people or man or men or woman or women)) or centenarian* or nonagenarian* or octogenarian* or septuagenarian* or sexagenarian* or dottering or decrepit or tottering or overaged or "oldest old" or supercentenarian*).tw,kf. | 415,057 |
| 4 | 1 or 2 or 3 | 3,541,381 |
| 5 | assisted living facilities/ or homes for the aged/ | 16,017 |
| 6 | independent living/ or community dwelling.mp. | 32,540 |
| 7 | Home Nursing/ | 8,658 |
| 8 | Long-term care/ | 27,887 |
| 9 | exp Nursing Homes/ | 43,354 |
| 10 | (nursing adj2 home?).tw,kf. | 37,668 |
| 11 | assisted living facilit*.tw,kf. | 899 |
| 12 | long-term care.tw,kf. | 25,151 |
| 13 | ((aged or home or domiciliary) adj2 care).tw,kf. | 35,276 |
| 14 | ageing in place?.tw,kf. | 209 |
| 15 | 5 or 6 or 7 or 8 or 9 or 10 or 11 or 12 or 13 or 14 | 157,732 |
| 16 | exp Computers/ | 84,453 |
| 17 | Technology/ | 15,380 |
| 18 | (digital adj2 (technolog* or electronic?)).tw,kf. | 6,421 |
| 19 | Self-Help Devices/ | 5,543 |
| 20 | Smart home?.tw,kf. | 922 |
| 21 | exp Wireless Technology/ | 4,448 |
| 22 | (Smart Devices or medical devices).mp. | 14,967 |
| 23 | Accidental Falls/pc [Prevention & Control] | 10,355 |
| 24 | Mobility Limitation/ | 5,194 |
| 25 | Mobile Applications/ or Telemedicine/ or Cell Phone/ | 50,459 |
| 26 | 16 or 17 or 18 or 19 or 20 or 21 or 22 or 23 or 24 or 25 | 188,294 |
| 27 | Outcome measures.mp. or Outcome Assessment, Health Care/ | 268,544 |
| 28 | implementation science/ | 1,145 |
| 29 | Behavioural indicator.mp. | 26 |
| 30 | Self Report/ | 41,172 |
| 31 | 27 or 28 or 29 or 30 | 308,345 |
| 32 | 4 and 15 and 26 and 31 | 472 |

exp Aged/
exp Geriatrics/
(elders or elderly or geriatric* or "gerontolog* old age" or (seniors not "high school") or (older adj3 (adult*or person* or people or man or men or woman or women)) or centenarian* or nonagenarian* or octogenarian* or septuagenarian* or sexagenarian* or dottering or decrepit or tottering or overaged or "oldest old" or supercentenarian*).tw,kf.
1 or 2 or 3
assisted living facilities/ or homes for the aged/
independent living/ or community dwelling.mp.
Home Nursing/
Long-term care/
exp Nursing Homes/
(nursing adj2 home?).tw,kf.
assisted living facilit*.tw,kf.
long-term care.tw,kf.
((aged or home or domiciliary) adj2 care).tw,kf.
ageing in place?.tw,kf.
5 or 6 or 7 or 8 or 9 or 10 or 11 or 12 or 13 or 14
exp Computers/
Technology/
(digital adj2 (technolog* or electronic?)).tw,kf.
Self-Help Devices/
Smart home?.tw,kf.
exp Wireless Technology/
(Smart Devices or medical devices).mp.
Accidental Falls/pc [Prevention & Control]
Mobility Limitation/
Mobile Applications/ or Telemedicine/ or Cell Phone/
16 or 17 or 18 or 19 or 20 or 21 or 22 or 23 or 24 or 25
Outcome measures.mp. or Outcome Assessment, Health Care/
implementation science/
Behavioural indicator.mp.
Self Report/
27 or 28 or 29 or 30
4 and 15 and 26 and 31

**Scopus Search Strategy – 1,752 Results [In Covidence]**

( TITLE-ABS-KEY ( ( outcome AND measures ) OR ( self AND report ) OR ( self AND assessment ) OR ( behavioural AND indicators ) ) ) AND ( TITLE-ABS-KEY ( computers OR technology OR ( electronic AND devices ) OR ( smart AND home ) OR ( self-help AND devices ) OR ( wireless AND technology ) OR ( smart AND devices ) OR ( mobile AND applications ) OR telemedicine ) ) AND ( TITLE-ABS-KEY ( aged OR elders OR elderly OR geriatric* ) ) AND ( TITLE-ABS-KEY ( ( assisted AND living AND facilit* ) OR ( independent AND living ) OR ( community AND dwelling ) OR ( home AND nursing ) OR ( nursing AND home ) OR ( long-term AND care ) ) ) AND ( LIMIT-TO ( DOCTYPE , "ar" ) OR LIMIT-TO ( DOCTYPE , "re" ) ) AND ( LIMIT-TO ( LANGUAGE , "English" ) ) AND ( LIMIT-TO ( SRCTYPE , "j" ) )

**PsycINFO – 10 Results [in Covidence]**

| **#** | **Query** | **Results from 3 Aug 2022** |
| --- | --- | --- |
| 1 | exp Aged/ | 2,806 |
| 2 | exp Geriatrics/ | 14,790 |
| 3 | (independent living or community dwelling).mp. | 15,590 |
| 4 | exp Long-term care/ | 5,997 |
| 5 | exp Nursing Homes/ | 9,680 |
| 6 | exp Computers/ | 47,994 |
| 7 | exp Technology/ | 256,616 |
| 8 | (Smart Devices or medical devices).mp. | 637 |
| 9 | Mobile Applications/ or Telemedicine/ or Cell Phone/ | 13,371 |
| 10 | Outcome measures.mp. or Outcome Assessment, Health Care/ | 31,944 |
| 11 | Behavioural indicator.mp. | 31 |
| 12 | Self Report/ | 21,409 |
| 13 | (Old age or elderly primipara).mp. | 15,287 |
| 14 | ("Housing for the Elderly" or "Homes for the Aged").mp. | 3,234 |
| 15 | Self Help Devices.mp. | 951 |
| 16 | (Indicators or Self Report).mp. | 154,867 |
| 17 | 1 or 2 or 13 | 31,501 |
| 18 | 3 or 4 or 5 or 14 | 31,309 |
| 19 | 6 or 7 or 8 or 9 or 15 | 267,671 |
| 20 | 10 or 11 or 12 or 16 | 183,828 |
| 21 | 17 and 18 and 19 and 20 | 10 |

exp Aged/
exp Geriatrics/
(independent living or community dwelling).mp.
exp Long-term care/
exp Nursing Homes/
exp Computers/
exp Technology/
(Smart Devices or medical devices).mp.
Mobile Applications/ or Telemedicine/ or Cell Phone/
Outcome measures.mp. or Outcome Assessment, Health Care/
Behavioural indicator.mp.
Self Report/
(Old age or elderly primipara).mp.
("Housing for the Elderly" or "Homes for the Aged").mp.
Self Help Devices.mp.
(Indicators or Self Report).mp.
1 or 2 or 13
3 or 4 or 5 or 14
6 or 7 or 8 or 9 or 15
10 or 11 or 12 or 16
17 and 18 and 19 and 20

**Embase – 370 Results [in Covidence]**

| **#** | **Query** | **Results from 3 Aug 2022** |
| --- | --- | --- |
| 1 | exp Aged/ | 3,407,136 |
| 2 | exp Geriatrics/ | 40,007 |
| 3 | (elders or elderly or geriatric* or "gerontolog* old age" or (seniors not "high school") or (older adj3 (adult*or person* or people or man or men or woman or women)) or centenarian* or nonagenarian* or octogenarian* or septuagenarian* or sexagenarian* or dottering or decrepit or tottering or overaged or "oldest old" or supercentenarian*).tw,kf. | 558,563 |
| 4 | 1 or 2 or 3 | 3,573,869 |
| 5 | assisted living facilities/ or homes for the aged/ | 13,504 |
| 6 | independent living/ | 6,533 |
| 7 | Home Nursing/ | 64,572 |
| 8 | Long-term care/ | 139,942 |
| 9 | exp Nursing Homes/ | 58,379 |
| 10 | (nursing adj2 home?).tw,kf. | 47,451 |
| 11 | assisted living facilit*.tw,kf. | 1,274 |
| 12 | long-term care.tw,kf. | 31,546 |
| 13 | ((aged or home or domiciliary) adj2 care).tw,kf. | 43,238 |
| 14 | 5 or 6 or 7 or 8 or 9 or 10 or 11 or 12 or 13 | 294,720 |
| 15 | exp Computers/ | 153,911 |
| 16 | Digital technology/ | 2,208 |
| 17 | (digital adj2 (technolog* or electronic?)).tw,kf. | 7,241 |
| 18 | Self-Help Devices/ | 2,216 |
| 19 | Smart Home*.mp. [mp=title, abstract, heading word, drug trade name, original title, device manufacturer, drug manufacturer, device trade name, keyword heading word, floating subheading word, candidate term word] | 952 |
| 20 | exp Wireless Technology/ | 6,757 |
| 21 | Smart Devices.mp. | 915 |
| 22 | exp Software/ | 281,007 |
| 23 | exp Aged/ | 3,407,136 |
| 24 | exp Geriatrics/ | 40,007 |
| 25 | (elders or elderly or geriatric* or "gerontolog* old age" or (seniors not "high school") or (older adj3 (adult*or person* or people or man or men or woman or women)) or centenarian* or nonagenarian* or octogenarian* or septuagenarian* or sexagenarian* or dottering or decrepit or tottering or overaged or "oldest old" or supercentenarian*).tw,kf. | 558,563 |
| 26 | 23 or 24 or 25 | 3,573,869 |
| 27 | assisted living facilities/ or homes for the aged/ | 13,504 |
| 28 | independent living/ or community dwelling.mp. | 41,477 |
| 29 | Home Nursing/ | 64,572 |
| 30 | Long-term care/ | 139,942 |
| 31 | exp Nursing Homes/ | 58,379 |
| 32 | (nursing adj2 home?).tw,kf. | 47,451 |
| 33 | assisted living facilit*.tw,kf. | 1,274 |
| 34 | long-term care.tw,kf. | 31,546 |
| 35 | ((aged or home or domiciliary) adj2 care).tw,kf. | 43,238 |
| 36 | ageing in place?.tw,kf. | 230 |
| 37 | 27 or 28 or 29 or 30 or 31 or 32 or 33 or 34 or 35 or 36 | 326,785 |
| 38 | exp Computers/ | 153,911 |
| 39 | Technology/ | 108,342 |
| 40 | (digital adj2 (technolog* or electronic?)).tw,kf. | 7,241 |
| 41 | Self-Help Devices/ | 2,216 |
| 42 | Smart home?.tw,kf. | 944 |
| 43 | exp Wireless Technology/ | 6,757 |
| 44 | (Smart Devices or medical devices).mp. | 20,934 |
| 45 | Accidental Falls/pc [Prevention & Control] | 2,842 |
| 46 | Mobility Limitation/ | 12,724 |
| 47 | Mobile Applications/ or Telemedicine/ or Cell Phone/ | 70,340 |
| 48 | 38 or 39 or 40 or 41 or 42 or 43 or 44 or 45 or 46 or 47 | 367,588 |
| 49 | Outcome measures.mp. or Outcome Assessment, Health Care/ | 687,023 |
| 50 | implementation science/ | 2,968 |
| 51 | Behavioural indicator.mp. | 35 |
| 52 | Self Report/ | 141,625 |
| 53 | 49 or 50 or 51 or 52 | 820,687 |
| 54 | 26 and 37 and 48 and 53 | 370 |

exp Aged/
exp Geriatrics/
(elders or elderly or geriatric* or "gerontolog* old age" or (seniors not "high school") or (older adj3 (adult*or person* or people or man or men or woman or women)) or centenarian* or nonagenarian* or octogenarian* or septuagenarian* or sexagenarian* or dottering or decrepit or tottering or overaged or "oldest old" or supercentenarian*).tw,kf.
1 or 2 or 3
assisted living facilities/ or homes for the aged/
independent living/
Home Nursing/
Long-term care/
exp Nursing Homes/
(nursing adj2 home?).tw,kf.
assisted living facilit*.tw,kf.
long-term care.tw,kf.
((aged or home or domiciliary) adj2 care).tw,kf.
5 or 6 or 7 or 8 or 9 or 10 or 11 or 12 or 13
exp Computers/
Digital technology/
(digital adj2 (technolog* or electronic?)).tw,kf.
Self-Help Devices/
Smart Home*.mp. [mp=title, abstract, heading word, drug trade name, original title, device manufacturer, drug manufacturer, device trade name, keyword heading word, floating subheading word, candidate term word]
exp Wireless Technology/
Smart Devices.mp.
exp Software/
exp Aged/
exp Geriatrics/
(elders or elderly or geriatric* or "gerontolog* old age" or (seniors not "high school") or (older adj3 (adult*or person* or people or man or men or woman or women)) or centenarian* or nonagenarian* or octogenarian* or septuagenarian* or sexagenarian* or dottering or decrepit or tottering or overaged or "oldest old" or supercentenarian*).tw,kf.
23 or 24 or 25
assisted living facilities/ or homes for the aged/
independent living/ or community dwelling.mp.
Home Nursing/
Long-term care/
exp Nursing Homes/
(nursing adj2 home?).tw,kf.
assisted living facilit*.tw,kf.
long-term care.tw,kf.
((aged or home or domiciliary) adj2 care).tw,kf.
ageing in place?.tw,kf.
27 or 28 or 29 or 30 or 31 or 32 or 33 or 34 or 35 or 36
exp Computers/
Technology/
(digital adj2 (technolog* or electronic?)).tw,kf.
Self-Help Devices/
Smart home?.tw,kf.
exp Wireless Technology/
(Smart Devices or medical devices).mp.
Accidental Falls/pc [Prevention & Control]
Mobility Limitation/
Mobile Applications/ or Telemedicine/ or Cell Phone/
38 or 39 or 40 or 41 or 42 or 43 or 44 or 45 or 46 or 47
Outcome measures.mp. or Outcome Assessment, Health Care/
implementation science/
Behavioural indicator.mp.
Self Report/
49 or 50 or 51 or 52
26 and 37 and 48 and 53

**Cochrane (Center for Controlled Trials) – 69 Results [in Covidence]**

| **#** | **Query** | **Results from 3 Aug 2022** |
| --- | --- | --- |
| 1 | exp Aged/ | 223,350 |
| 2 | exp Geriatrics/ | 215 |
| 3 | (elders or elderly or geriatric* or "gerontolog* old age" or (seniors not "high school") or (older adj3 (adult*or person* or people or man or men or woman or women)) or centenarian* or nonagenarian* or octogenarian* or septuagenarian* or sexagenarian* or dottering or decrepit or tottering or overaged or "oldest old" or supercentenarian*).tw,kf. | 63,512 |
| 4 | 1 or 2 or 3 | 270,421 |
| 5 | assisted living facilities/ or homes for the aged/ | 721 |
| 6 | independent living/ or community dwelling.mp. | 5,435 |
| 7 | Home Nursing/ | 279 |
| 8 | Long-term care/ | 1,182 |
| 9 | exp Nursing Homes/ | 1,542 |
| 10 | (nursing adj2 home?).tw,kf. | 4,389 |
| 11 | assisted living facilit*.tw,kf. | 108 |
| 12 | long-term care.tw,kf. | 1,818 |
| 13 | ((aged or home or domiciliary) adj2 care).tw,kf. | 3,726 |
| 14 | ageing in place?.tw,kf. | 4 |
| 15 | 5 or 6 or 7 or 8 or 9 or 10 or 11 or 12 or 13 or 14 | 15,541 |
| 16 | exp Computers/ | 2,094 |
| 17 | Technology/ | 140 |
| 18 | (digital adj2 (technolog* or electronic?)).tw,kf. | 413 |
| 19 | Self-Help Devices/ | 148 |
| 20 | Smart home?.tw,kf. | 12 |
| 21 | exp Wireless Technology/ | 48 |
| 22 | (Smart Devices or medical devices).mp. | 553 |
| 23 | Mobility Limitation/ | 554 |
| 24 | Mobile Applications/ or Telemedicine/ or Cell Phone/ | 4,140 |
| 25 | Outcome measures.mp. or Outcome Assessment, Health Care/ | 67,803 |
| 26 | implementation science/ | 56 |
| 27 | Self Report/ | 2,696 |
| 28 | 16 or 17 or 18 or 19 or 20 or 21 or 22 or 23 or 24 | 7,540 |
| 29 | 25 or 26 or 27 | 70,207 |
| 30 | 4 and 15 and 28 and 29 | 69 |

exp Aged/
exp Geriatrics/
(elders or elderly or geriatric* or "gerontolog* old age" or (seniors not "high school") or (older adj3 (adult*or person* or people or man or men or woman or women)) or centenarian* or nonagenarian* or octogenarian* or septuagenarian* or sexagenarian* or dottering or decrepit or tottering or overaged or "oldest old" or supercentenarian*).tw,kf.
1 or 2 or 3
assisted living facilities/ or homes for the aged/
independent living/ or community dwelling.mp.
Home Nursing/
Long-term care/
exp Nursing Homes/
(nursing adj2 home?).tw,kf.
assisted living facilit*.tw,kf.
long-term care.tw,kf.
((aged or home or domiciliary) adj2 care).tw,kf.
ageing in place?.tw,kf.
5 or 6 or 7 or 8 or 9 or 10 or 11 or 12 or 13 or 14
exp Computers/
Technology/
(digital adj2 (technolog* or electronic?)).tw,kf.
Self-Help Devices/
Smart home?.tw,kf.
exp Wireless Technology/
(Smart Devices or medical devices).mp.
Mobility Limitation/
Mobile Applications/ or Telemedicine/ or Cell Phone/
Outcome measures.mp. or Outcome Assessment, Health Care/
implementation science/
Self Report/
16 or 17 or 18 or 19 or 20 or 21 or 22 or 23 or 24
25 or 26 or 27
4 and 15 and 28 and 29
